# Unraveling novel therapeutic targets for diffuse large B-cell lymphoma: a plasma multi-omics study

**DOI:** 10.3389/fphar.2026.1833739

**Published:** 2026-04-30

**Authors:** Xu Sun, Kai Kang, Yijun Wu, Yuqi Yang, Laduona Wang, Yuhong Liu, Lanqing Huo, Ting Niu, Ailin Zhao

**Affiliations:** 1 Department of Hematology, Institute of Hematology, Center for High Altitude Medicine, West China Hospital, Sichuan University, Chengdu, Sichuan, China; 2 State Key Laboratory of Biotherapy, Collaborative Innovation Center of Biotherapy, West China Hospital, Sichuan University, Chengdu, Sichuan, China; 3 National Facility for Translational Medicine (Sichuan), West China Hospital, Sichuan University, Chengdu, Sichuan, China; 4 Division of Thoracic Tumor Multimodality Treatment, Cancer Center, West China Hospital, Sichuan University, Chengdu, Sichuan, China; 5 West China School of Medicine, Sichuan University, Chengdu, Sichuan, China; 6 Department of Radiation Oncology, State Key Laboratory of Oncology in South China, Sun Yat-sen University Cancer Center, Guangzhou, China

**Keywords:** diffuse large B-cell lymphoma, genome-wide association studies, plasma multi-omics, single-cell sequencing, therapeutic target, TUBB

## Abstract

**Background:**

Diffuse large B-cell lymphoma (DLBCL), the most common non-Hodgkin lymphoma, exhibits genetic heterogeneity and variable treatment responses. Discovering new therapeutic targets is critical to improve outcomes. Given the lack of plasma protein studies for DLBCL, this study aims to first identify novel therapeutic targets based on plasma multi-omics and genome-wide association studies with further verifications.

**Methods:**

By two-step training (N = 456,948 participants) and validation (N = 288,927 participants), druggable genes and proteins were identified through the mendelian randomization analyses of expression quantitative trait loci (eQTL) and protein quantitative trait loci (pQTL) using the largest DLBCL dataset of genome-wide association studies. Further verifications were conducted through colocalization analysis, risk factor and related disease analysis, single-cell analysis, *ex-vivo* experiments and transcriptomics.

**Results:**

A total of 16 genes, 2 cis- and 3 trans-regulated plasma proteins were found to be potential targets for DLBCL with significant risks. The colocalization analysis revealed that six genes (APOM, C4A, C4B, CYP21A2, HCP5 and NEU1) and two proteins (MICB and IL17F) shared the causal genetic variants with DLBCL, suggesting their potential as therapeutic targets. The single-cell analysis for DLBCL tumors confirmed their cell type-specific expressions, suggesting that TUBB, with high risk for DLBCL, was highly expressed in malignant B cells with expression levels increasing as the tumor malignancy worsens. Further experiments and transcriptomics using the TUBB inhibitor demonstrated its anti-DLBCL efficacy, highlighting the therapeutic promise of targeting TUBB.

**Conclusion:**

This study unravels a series of novel therapeutic targets that provide new insights into the development of DLBCL treatment.

## Introduction

Diffuse large B-cell lymphoma (DLBCL) remains to be the predominant subtype of non-Hodgkin’s lymphomas, known for its high incidence and invasiveness (Siegel et al., 2022; Smith et al., 2015). Despite the potentially curable regimen with rituximab, cyclophosphamide, doxorubicin, vincristine, and prednisone (R-CHOP), approximately 30%–40% of DLBCL patients would encounter recurrence or progress following the initial immunochemotherapy (Coiffier et al., 2010; Wang and Li, 2020; Liang et al., 2023; Wang et al., 2020). Therefore, it is highly desirable to explore novel drug targets for improving current therapeutic strategies of DLBCL (Ayyappan and Maddocks, 2019; Lu et al., 2023).

Human proteins are essential participants in numerous biological processes and represent a principal class of targets for new drug discovery (Zheng et al., 2020). Circulating plasma proteins present in the bloodstream, whether due to cellular leakage or active secretion, offer insight into human health status and specific diseases, and serve as significant reservoirs of biomarkers and potential targets for drug development (Suhre et al., 2021). Protein dysregulation is critical to lymphoma biology (Leeman-Neill et al., 2023). Although targeting some protein molecules or pathways such as B-cell receptor signaling and the downstream NF-κB and phosphatidylinositol 3-kinase (PI3K) cascades can be used to treat DLBCL, there has been little research exploring the potential of plasma proteins as therapeutic targets and biomarkers for this heterogeneous disease (Liu et al., 2021; Xu et al., 2021; Lumish et al., 2021; Decruyenaere et al., 2021; Sarode et al., 2025).

Current advancements in high-throughput genomics, transcriptomics and proteomics technologies have enabled the identification of numerous expression quantitative trait loci (eQTL) and protein quantitative trait loci (pQTL) through a series of large-scale omics studies, which provide a valuable resource for linking genetically associated protein targets with diseases, significantly increasing the likelihood of obtaining market approval of drugs developed through this approach (Nelson et al., 2015). A novel genetic instrumental variable analysis method called Mendelian Randomization (MR) has recently been developed, which utilizes single nucleotide polymorphisms (SNPs) from genome-wide association studies (GWAS) as genetic instruments to estimate the causal effects of exposures on outcomes (Davies et al., 2018). Compared to observational study methods, MR can effectively reveal causal relationships between exposures and diseases, minimizing the potential for reverse causation and confounding biases. MR analysis has facilitated the discovery of various disease etiologies and drug targets (Considine et al., 2021; Lin et al., 2023; Sun et al., 2023; Wingo et al., 2021). However, there are currently no reports of integrating multi-omics to explore protein targets and biomarkers in DLBCL.

**FIGURE 1:**
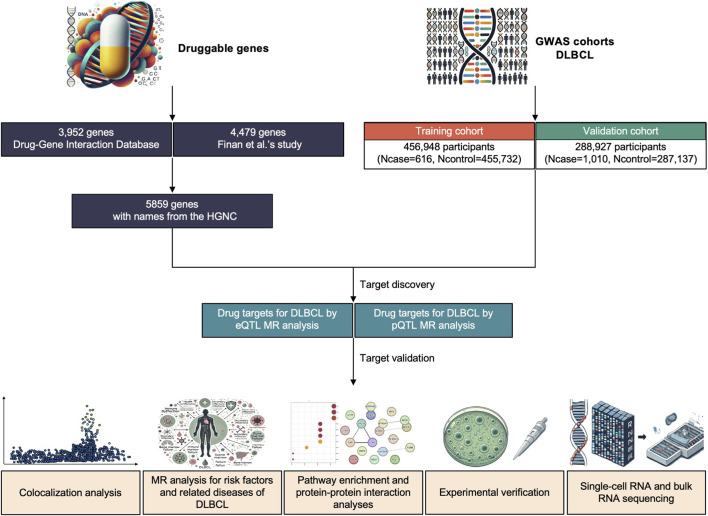
Flowchart of the study design. DLBCL, diffuse large B-cell lymphoma. HGNC, Human Genome Organization Gene Nomenclature Committee. eQTL, expression quantitative trait loci. pQTL protein quantitative trait loci. MR, Mendelian Randomization.

In the present study, we utilized multiple methods, which involved eQTL, pQTL, colocalization analysis, etc., to identify novel potential drug targets for DLBCL by integrating plasma proteomics and transcriptomes with multi-origin genetic data. Further verification of the critical targets was performed by analyzing single-cell transcriptomes and conducting validation experiments (Figure 1).

## Methods

### Acquisition of druggable gene set

Potentially druggable genes were obtained from the Finan et al.’s study (Finan et al., 2017) and Drug-Gene Interaction Database (DGIdb V.4.2.0, https://www.dgidb.org/), which provides information on drug-gene interactions and druggable genes through publications, databases and other online sources.

### GWAS summary statistics of DLBCL

For the primary analysis, the GWAS data on DLBCL (GCST90043906) was obtained from the GWAS Catalog database (https://www.ebi.ac.uk/gwas/), including 456,948 participants (N_case_ = 616, N_control_ = 455,732) of European Ancetry. For the external validation, the summary GWAS statistics of DLBCL (finngen_R9 _C3_DLBC L_EXALLC) was retrieved from the FinnGen database (https://r9.finngen.fi/) that enrolled 288,927 individuals (N_case_ = 1,010, N_control_ = 287,137).

### GWAS summary statistics for risk factors and associated diseases of DLBCL

A series of GWAS summary datasets for risk factors (chronic hepatitis, obesity, HIV infection and autoimmune diseases) and associated diseases (Hodgkin lymphoma, follicular lymphoma and multiple myeloma) of DLBCL were retrieved from the GWAS Catalog database.

### pQTL and eQTL data acquisition

We utilized 4,118 pQTLs of plasma proteome derived from a large-scale proteogenomic study by Alexander et al. that involved 5,368 individuals in Iceland, which revealed 4,035 independent associations between genetic variants and 2,091 plasma proteins (Gudjonsson et al., 2022). The majority of cis-pQTL and trans-pQTL are unique to individual proteins.

The summary statistics of eQTL data were sourced from eQTLGen Consortium (https://eqtlgen.org/). The cis-eQTLs for 16,989 genes were obtained from 31,684 blood samples of healthy individuals of European descent, involving significant cis-eQTLs’ results (false discovery fate, FDR < 0.05) and allele frequency information.

### MR analysis for eQTL/pQTL and DLBCL

The identified genes with plasma cis-eQTL data (FDR < 0.05) were obtained by intersecting the gene set from the eQTLGen Consortium with the potential druggable gene set. These cis-eQTL genetic instrument variables were selected through linkage disequilibrium (LD) analysis (LD clumping r^2^ at 0.01, genetic distance at 10,000 kb), ensuring the reliability of our MR analysis. Then, we performed two-sample MR analyses with the druggable targets at gene expression as the exposure and DLBCL as the outcome. Similarly, two-sample MR analyses were also conducted with potential druggable proteins as the exposure and DLBCL as the outcome. For all MR analyses, five methods were employed: inverse-variance weighted (IVW) test, weighted mode, MR-Egger regression, Simple mode and Weighted median. IVW method was reported to perform slightly better than others under certain conditions. Therefore, results with more than one instrumental variable primarily rely on the IVW method, with the other four methods serving as complements. The Wald Ratio method was applied when there was only one significant instrument variable.

### Bayesian colocalization analysis

The Bayesian testing was performed to enhance the MR findings, employing the R package coloc (version 5.2.3) for estimating the posterior probability of shared variants (Wallace, 2021). In the MR analysis, the lead SNP is defined as the instrumental variable with the smallest p-value. For each significant MR finding, all SNPs positioned within 200 kb upstream and downstream of each lead SNP were gathered for the Bayesian colocalization analysis between QTLs and the GWAS. In the R package coloc, default values of prior probabilities were employed: P1 = 1 × 10^−4^, P2 = 1 × 10^−4^, P12 = 1 × 10^−5^. Here, P1 represented the probability of a given SNP being associated with DLBCL; P2 represented the probability of a given SNP being a significant eQTL or pQTL; and P12 represented the probability of a given SNP being an outcome of both DLBCL and eQTL/pQTL. This Bayesian model assumes that each trait within a test region has at most one association, and calculates posterior probabilities for five hypotheses (PPH0-PPH4) on whether a single variant is shared between eQTL/pQTL and DLBCL: PPH0, no correlation with either trait; PPH1, correlated with eQTL or pQTL, unrelated to the risk of DLBCL; PPH2, correlated with the risk of DLBCL, unrelated to eQTL or pQTL; PPH3, correlated with both eQTL/pQTL and the risk of DLBCL, with distinct causal variants; PPH4: correlated with both eQTL/pQTL and the risk of DLBCL, with a shared causal variant. A higher PPH4 value was considered strong support for colocalization, using the default settings of prior probabilities in the R package coloc. Due to the limited capacity of the colocalization analysis, we focused our analysis on genes or proteins with a combined PPH3+PPH4 score of ≥0.8.

### MR analysis of risk factors and related diseases of DLBCL using eQTL and pQTL

Based on the results of colocalization analysis, another set of two-sample MR analyses with similar parameters was conducted with the druggable genes or proteins (both cis-pQTL and trans-pQTL) that were positively identified in the colocalization analysis as the exposure and the risk factors and related diseases of DLBCL as the outcome.

### Pathway enrichment and protein-protein interaction analyses

Pathway enrichment analysis, based on Gene Ontology (GO) and Kyoto Encyclopedia of Genes and Genomes (KEGG) terms, was conducted using the R package clusterProfiler on the genes identified through MR analysis (Wu et al., 2021). Protein-protein interaction analysis was performed based on the STRING database (https://string-db.org/).

### Single-cell RNA sequencing analysis

The data of scRNA-seq and corresponding clinical characteristics were obtained from two publicly available datasets. The dataset reported by Steen et al. is available under accession number GSE182434 in the Gene Expression Omnibus (GEO) database (https://www.ncbi.nlm.nih.gov/geo/). The dataset reported by Roider et al. is available under accession number VRJUNV in the heiDATA database (https://heidata.uni-heidelberg.de/). When merging the two datasets, we retained only the genes that were present in both datasets. We then processed the data using the standard Seurat (version 4.1.0) pipeline (Hao et al., 2021). First, we normalized the count-format expression matrix. The feature counts for each cell were divided by the total counts for that cell and multiplied by a scale factor of 10,000, followed by a log transformation using log1p. We then identified highly variable genes using the FindVariableFeatures function, followed by scaling the data with ScaleData and using the scaled data to perform PCA. Uniquely, after PCA, we chose to run Harmony (version 0.1.0) on the top 50 principal components to remove batch effects arising from different dataset sources (Korsunsky et al., 2019). The subsequent clustering and dimensionality reduction were then based on the Harmony-adjusted results.

The differential expression analysis of the scRNA-seq was using the R package MAST (version 1.20.0) (Finak et al., 2015). The pseudotime analysis was using the R package monocle (version 2.22.0) (Qiu et al., 2017). The fit curve between gene expression levels and pseudotimes was generated using the locally estimated scatterplot smoothing (LOESS) method, with a 95% confidence interval plotted.

### Western blotting

Protein lysates were extracted using RIPA lysis buffer supplemented with protease and phosphatase inhibitors (MCE, HY-K0021 and K0010). After quantification and normalization with a bicinchoninic acid (BCA) protein assay kit (EpiZyme, ZJ102, Shanghai, China), 20 μg of total protein was subjected to electrophoresis on 12.5% polyacrylamide gels (EpiZyme, PG213). The separated proteins were then transferred onto polyvinylidene fluoride (PVDF) membranes and blocked with protein-free rapid blocking buffer (EpiZyme, PS108P) for 30 min at room temperature. Subsequently, the membranes were incubated with anti-GAPDH (Abcam, ab8245, 1/5,000), anti-β-Tubulin (CST, 2,146, 1/1,000) and anti-cleaved caspase-3 (CST, 9,661, 1/1,000) at 4 °C overnight. Secondary antibodies HRP-linked anti-rabbit IgG (Bio X cell, BX-2301, 1/5,000) were detected using the e-BLOT Touch Imager (e-BLOT, Shanghai, China).

### Apoptosis detection

Two human DLBCL cell lines (U2932 and SU-DHL-6) were used. Cells (5 × 10^5^) cultured in 6-well plates treated with DMSO or 1 μM ABT-751 for 24 h and 48 h were collected and incubated with Annexin V-PI staining solution (Beyotime, C1062S, Shanghai, China) for 15 min at 4 °C after washing with cold PBS twice. Three independent experiments were performed. Data were obtained on a CytoFLEX Flow Cytometer (Beckman) and analyzed with CytExpert (v.2.4).

### Bulk RNA sequencing analysis

RNA extractions were performed on *in vitro* cultured cells using the TRIzol reagent (Invitrogen, CA, United States) following the manufacturer’s instructions. RNA purity was evaluated with a NanoDrop 2000 spectrophotometer (Thermo Scientific, United States), while RNA integrity was assessed using the Agilent 2,100 Bioanalyzer (Agilent Technologies, Santa Clara, CA, United States). Then the libraries were prepared using the VAHTS Universal V6 RNA-seq Library Prep Kit. Sequencing was then conducted on the Illumina Novaseq 6,000 platform and 150 bp paired-end reads were generated. Raw reads of fastq format were firstly processed using fastp (version 0.23.4) and the low-quality reads were removed to obtain the clean reads (Chen, 2023). The reads were aligned to the human reference genome (GRCh38) using HISAT2 (version 2.2.1) (Kim et al., 2019). Gene read counts were obtained with HTSeq-count (version 0.9.1). The count-format expression data were used for differential expression analysis with the R package edgeR (version 3.36.0) (Chen et al., 2016). Genes with a fold change greater than or equal to 1.5 and an adjusted p-value of less than 0.05 were defined as differentially expressed genes and were subsequently used for pathway enrichment analysis based on GO terms. The count-format expression data were converted to transcripts per million (TPM) formats, followed by a binary logarithmic transformation using the log1p function. The transformed TPM data were then used for PCA. Additionally, GSVA and PROGENy analyses were performed using the GSVA (version 1.42.0) and progeny (version 1.16.0) R packages, respectively (Hänzelmann et al., 2013; Schubert et al., 2018).

## Results

### Druggable genes

A total of 3,952 potentially druggable genes (Supplementary Table 1) were obtained from the Drug-Gene Interaction Database, and another set of 4,479 druggable genes (Supplementary Table 2) was retrieved from Finan et al.’s study (Finan et al., 2017). Finally, a list of 5,859 unique druggable genes with names from the Human Genome Organization Gene Nomenclature Committee (HGNC) were compiled based on the two sources for further analysis (Supplementary Table 3).

### Drug targets for DLBCL by eQTL MR analysis

**FIGURE 2:**
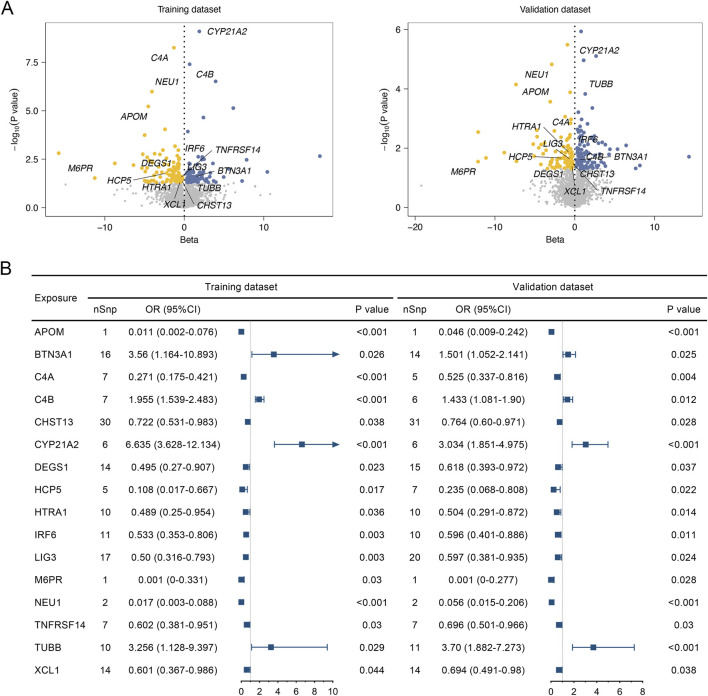
Associations of the identified risk genes with diffuse large B-cell lymphoma (DLBCL) determined by eQTL Mendelian Randomization analyses. **(A)** Volcano plot of individual druggable genes associated with the DLBCL outcome in the training dataset (GCST90043906) and validation dataset (finngen_R9_C3_DLBCL_EXALLC). **(B)** Forest plot of candidate druggable genes that demonstrated significant associations with DLBCL risk in both the training and validation datasets. nSnp, number of single nucleotide polymorphisms. OR, odds ratio.

Firstly, the gene list from the eQTLGen Consortium was intersected with the druggable gene set in Supplementary Table 3, and thus a total of 3,724 potential genes with the eQTL data (FDR<0.05) were identified for MR analysis (LD clumping r^2^ at 0.01, genetic distance at 10,000 kb). We then performed MR analyses with the eQTL as the exposure and DLBCL as the outcome, identifying 193 potential druggable genes (Supplementary Table 4) in the training set (GCST90043906) and 214 potential druggable genes (Supplementary Table 5) for DLBCL in the validation set (finngen_R9_C3_DLBCL_EXALLC) by the inverse-variance weighted (IVW) or the Wald ratio method. Thus, 16 candidate druggable genes were finally verified that can be potential targets for DLBCL (Figure 2).

### Drug targets for DLBCL by pQTL MR analysis

**FIGURE 3:**
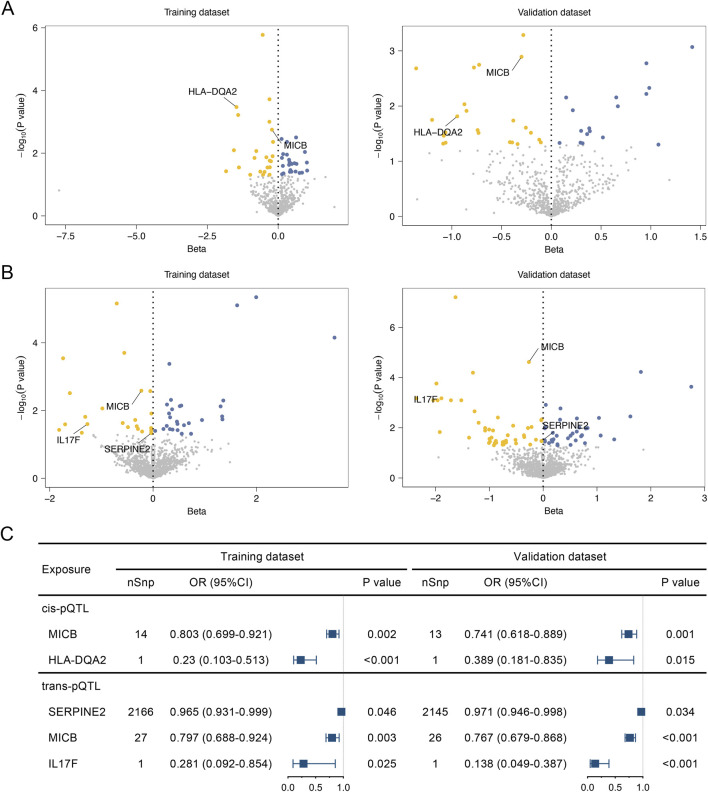
Associations of the identified risk plasma proteins with diffuse large B-cell lymphoma (DLBCL) determined by pQTL Mendelian Randomization analyses. **(A)** Volcano plot of individual druggable proteins associated with the DLBCL outcome by cis-pQTL Mendelian Randomization (MR) analyses in the training dataset (GCST90043906) and validation dataset (finngen_R9_C3_DLBCL_EXALLC). **(B)** Volcano plot of individual druggable proteins associated with the DLBCL outcome by trans-pQTL MR analyses in the training dataset and validation dataset. **(C)** Forest plot of candidate druggable proteins that demonstrated significant associations with DLBCL risk in both the training and validation datasets. nSnp, number of single nucleotide polymorphisms. OR, odds ratio.

Based on the loci information of druggable genes, the pQTL data can be classified into cis-pQTL (P < 1 × 10^−5^; within 1 mb of the gene region) and trans-pQTL (P < 5 × 10^−8^; beyond 1 mb of the gene region). Instrumental variables for each gene were identified through LD analysis for both cis-pQTL and trans-pQTL (LD clumping r^2^ at 0.01, genetic distance at 10,000 kb). MR analyses were conducted with cis-pQTL and trans-pQTL separately as the exposure and DLBCL as the outcome. The results demonstrated 46 (Supplementary Table 6) and 40 (Supplementary Table 7) potential protein targets for cis-pQTL in the training (GCST90043906) and validation (finngen_R9_C3_DLBCL_EXALLC) datasets, respectively. In the MR results of trans-pQTL with DLBCL, 50 candidate protein targets (Supplementary Table 8) were identified in the training dataset, while 81 candidates (Supplementary Table 9) were in the validation dataset. Finally, there were two (MICB, HLA-DQA2) and three (SERPINE2, MICB, IL17F) proteins verified successfully that can be druggable by the cis-pQTL and trans-pQTL MR analyses, respectively (Figure 3).

### Colocalization analysis

Based on the MR results, a Bayesian colocalization test was conducted on the lead SNPs of candidate drug targets verified both in the training and validation analyses to determine the probability that these SNPs associated with DLBCL and eQTL/pQTL shared causal genetic variants (colocalization). For prior genes, *APOM, C4A, C4B, CYP21A2, HCP5* and *NEU1* showed positive results (PPH3+PPH4 >0.8) in both the training and validation datasets (Table 1). As for the plasma proteome analysis, MICB demonstrated positive results for both cis- and trans-pQTL in both datasets, while IL17F was only for trans-pQTL.

**TABLE 1:** Results of colocalization analysis in the training and validation datasets.

Target	Dataset	nSnp	PPH0	PPH1	PPH2	PPH3	PPH4	PPH3+PPH4
Gene								
APOM	finngen_R9_C3_DLBCL_EXALLC	438	1.00E-304	0.118	6.42E-304	0.758	0.123	0.882
C4A	finngen_R9_C3_DLBCL_EXALLC	390	9.73E-305	0.027	3.46E-303	0.950	0.023	0.973
C4B	finngen_R9_C3_DLBCL_EXALLC	439	9.85E-305	0.116	6.46E-304	0.763	0.121	0.884
CYP21A2	finngen_R9_C3_DLBCL_EXALLC	380	9.73E-305	0.027	3.46E-303	0.950	0.023	0.973
HCP5	finngen_R9_C3_DLBCL_EXALLC	696	2.94E-304	0.126	1.80E-303	0.769	0.106	0.874
NEU1	finngen_R9_C3_DLBCL_EXALLC	438	1.00E-304	0.118	6.42E-304	0.758	0.123	0.882
APOM	GCST90043906	458	4.59E-305	0.057	2.59E-304	0.318	0.625	0.943
C4A	GCST90043906	400	6.66E-305	0.018	3.90E-304	0.103	0.879	0.982
C4B	GCST90043906	459	4.59E-305	0.057	2.59E-304	0.318	0.625	0.943
CYP21A2	GCST90043906	388	6.66E-305	0.018	3.90E-304	0.103	0.879	0.982
HCP5	GCST90043906	745	7.01E-305	0.034	3.19E-304	0.152	0.814	0.966
NEU1	GCST90043906	458	4.59E-305	0.057	2.59E-304	0.318	0.625	0.943
Protein								
IL17F_trans	finngen_R9_C3_DLBCL_EXALLC	711	7.05E-86	0.021	3.02E-84	0.902	0.077	0.979
IL17F_trans	GCST90043906	778	1.77E-273	0.020	8.51E-272	0.975	0.005	0.980
MICB_trans	finngen_R9_C3_DLBCL_EXALLC	4,815	4.30E-284	0.067	5.91E-283	0.929	0.004	0.933
MICB_cis	finngen_R9_C3_DLBCL_EXALLC	1,891	3.56E-284	0.147	2.04E-283	0.844	0.009	0.853
MICB_trans	GCST90043906	5,238	3.03E-283	0.005	5.89E-281	0.995	0.000	0.995
MICB_cis	GCST90043906	1,965	9.61E-285	0.041	2.24E-283	0.945	0.015	0.959

Training dataset: GCST90043906; validation dataset: finngen_R9_C3_DLBCL_EXALLC. nSnp: the number of SNPs. PPH0-PPH4: posterior probabilities for five hypotheses on whether a single variant is shared between eQTL/pQTL and DLBCL; PPH0, no correlation with either trait; PPH1, correlated with eQTL or pQTL, unrelated to the risk of DLBCL; PPH2, correlated with the risk of DLBCL, unrelated to eQTL or pQTL; PPH3, correlated with both eQTL/pQTL and the risk of DLBCL, with distinct causal variants; PPH4: correlated with both eQTL/pQTL and the risk of DLBCL, with a shared causal variant. PPH4 ≥ 0.8 is generally considered strong support for a shared causal variant. PH3 + PPH4 ≥ 0.8 indicates overall colocalization (i.e., both traits share at least one causal variant in the region), with higher PPH4 relative to PPH3 favoring shared causality. In this table, all listed genes and proteins show PPH3+PPH4 > 0.85, and several (e.g., C4A, CYP21A2, IL17F) have PPH4 > 0.8 in at least one dataset, suggesting that their association with DLBCL, is likely driven by the same genetic variants that regulate their expression or protein levels.

### MR analysis for risk factors and related diseases of DLBCL

**FIGURE 4:**
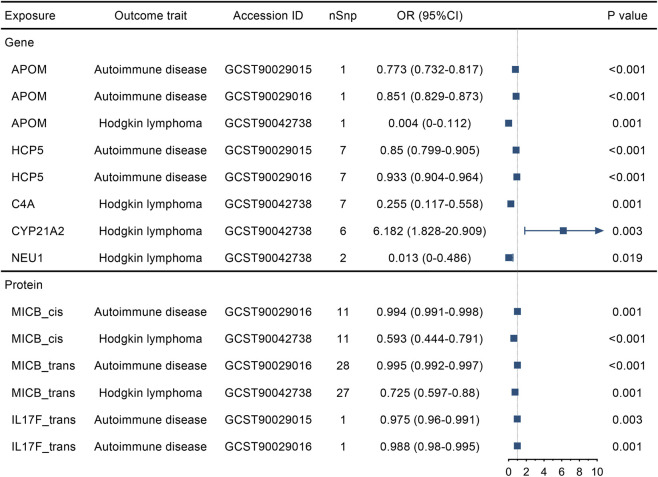
Associations of the identified candidate druggable genes and proteins with risk factors and related diseases of diffuse large B-cell lymphoma determined by Mendelian Randomization analyses. nSnp, number of single nucleotide polymorphisms. OR, odds ratio.

To explore the feasibility of inferring the risk of DLBCL through the detection of gene expression or plasma protein levels, we conducted additional MR analyses. The eQTLs or pQTLs for candidate targets, identified positively in the colocalization analysis, were used as the exposure, while the risk factors and related diseases of DLBCL (Supplementary Table 10) were considered as the outcome. The results demonstrated that autoimmune diseases and Hodgkin lymphoma met the significant associations with some candidate targets of genes or proteins (Figure 4). For example, the elevated expression of APOM, which reduced the risk of DLBCL (Figure 2), also reduced the risk of its risk factor, autoimmune diseases (GCST90029015, OR: 0.773, 95%CI: 0.732–0.817, P < 0.001; GCST90029016, OR: 0.851, 95%CI: 0.829–0.873, P < 0.001), and the associated disease, Hodgkin lymphoma (GCST90042738, OR: 0.004, 95%CI: 0–0.112, P = 0.001). All significant results regarding risk factors or related diseases for DLBCL were consistent with the above observed MR analyses of the DLBCL risk.

### Resolve cell type-specific expression at single-cell resolution

**FIGURE 5:**
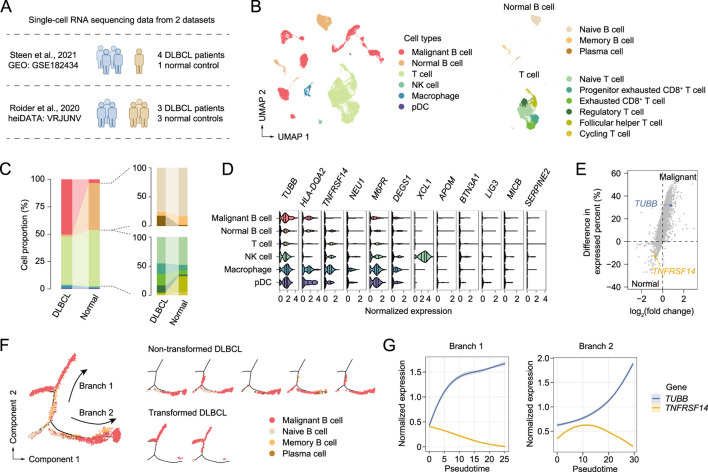
Single-cell RNA sequencing in DLBCL for the genes identified by MR analyses. **(A)** The collection of scRNA-seq data from DLBCL patients and normal controls. **(B)** Uniform manifold approximation and projection (UMAP) plots of cells from scRNA-seq of DLBCL patients and normal controls, colored by cell type. **(C)** Bar plots showing the proportion of each cell type, colored by cell type. For the cell type color code, see **(B)**. **(D)** Violin plots showing the normalized expression levels of genes identified by MR analyses in different cell types, with 25th, 50th and 75th percentiles marked. **(E)** Scatter plot showing the differentially expressed genes between malignant and normal B cells. Two representative genes are labeled. **(F)** The branched trajectory of B cell transition in a two-dimensional state-space inferred by Monocle. Cells are shown as a whole (left), and cells of each DLBCL patients are shown separately (right). **(G)** Fitted curve representing the trend of gene expression levels over pseudotime. The shaded area denotes the 95% confidence interval around the fitted line.

Based on the identified potentially druggable genes obtained from the MR analysis, we conducted pathway enrichment and protein-protein interaction analyses, which, however, revealed no obvious interactions among these genes (Supplementary Figure 1; Supplementary Figure 2). This finding prompted us to investigate the cell type-specific expression of these genes at single-cell resolution. We collected single-cell RNA sequencing (scRNA-seq) data from two publicly available datasets of DLBCL (Roider et al., 2020; Steen et al., 2021), which, after integration, comprised 24,418 cells from 7 DLBCL patients and 11,565 cells from 4 normal controls (Figure 5A). Based on cell-specific marker genes (Supplementary Figure 3), we categorized these cells into malignant B cells, normal B cells, T cells, NK cells, macrophages, and plasmacytoid dendritic cells (pDCs). Further classification of normal B cells identified naive B cells, memory B cells, and plasma cells, while T cells were subdivided into naive T cells, progenitor exhausted CD8^+^ T cell, exhausted CD8^+^ T cells, regulatory T cells (Tregs), follicular helper T cells (Tfhs), and cycling T cells (Figure 5B). By comparing the differences in cell proportions between the two groups, we observed a higher proportion of plasma cells and a lower proportion of memory B cells in DLBCL (Figure 5C). The increased presence of Tregs and exhausted CD8^+^ T cells suggested an immunosuppressive tumor microenvironment in DLBCL (Figure 5C).

Regarding cell type-specific gene expression, we focused on previously identified candidate genes that were expressed in at least 1% of cells. *XCL1* was found to exhibit highly specific expression in NK cells, while HLA-DQA2, an MHC-II encoding gene, is expressed in antigen-presenting macrophages, pDCs, and B cells (Figure 5D). Notably, *TUBB* is highly expressed in malignant B cells, whereas *TNFRSF14* shows the opposite pattern (Figure 5E). Furthermore, we performed pseudotime analysis on B cells and identified two distinct trajectories of differentiation from normal to malignant B cells (Figure 5F). Interestingly, branch 1 corresponds to DLBCL samples derived from non-transformed origins, while branch 2 aligns with DLBCL samples derived from follicular lymphoma (FL) transformation. However, regardless of the trajectory, the expression level of the *TUBB* gene in B cells consistently increases over pseudotime, further demonstrating the crucial role of *TUBB* in DLBCL (Figure 5G).

### Potential druggability of targeting *TUBB* in DLBCL

**FIGURE 6:**
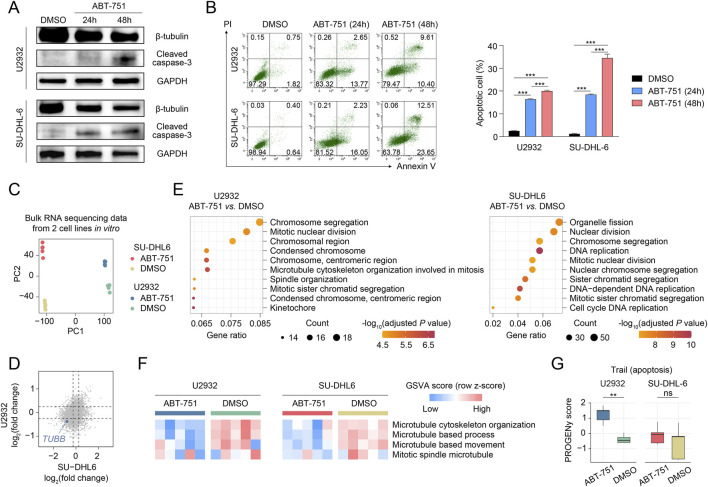
**(A)** The western blot showing the protein expression of β-Tubulin and cleaved caspase-3 in U2932 cells and SU-DHL-6 cells treated with 1 μM ABT-751 for 24 h and 48 h. **(B)** Apoptotic proportion and representative flow cytometry plots of U2932 cells and SU-DHL-6 cells treated with 1 μM ABT-751 for 24 h and 48 h as detected by annexin V-PI-based flow cytometry. The statistical analysis was performed using one-way ANOVA with Tukey’s multiple comparisons test. P values are shown and error bars indicate mean value ± standard deviation. **(C)** PCA of all samples based on the bulk RNA-seq. **(D)** Scatter plot showing the differentially expressed genes between ABT-751 and DMSO groups of two cell lines. **(E)** Pathway enrichment analysis of differentially expressed genes between ABT-751 and DMSO groups of two cell lines. **(F)** Heatmaps showing the GSVA scores of representative microtubule-related pathways in each sample. **(G)** Boxplot showing PROGENy scores of apoptosis pathway in each sample. The centerline indicates the median value, the lower and upper hinges represent the 25th and 75th percentiles, respectively, and the whiskers extend to 1.5 times the interquartile range. *P* values were determined by the two-sided Wilcoxon rank-sum test. *P value < 0.05, ** P value < 0.01, *** P value < 0.001.

*TUBB* is a class of microtubule protein-coding genes that form the basic structure of the cellular microtubule skeleton, responsible for many important functions such as cell movement and cell division (Gudimchuk and McIntosh, 2021; Janakiraman et al., 2023). Due to the potential anti-tumor effects of microtubule-targeting drugs, TUBB has also been found to be associated with various clinical and biological characteristics of multiple cancers, making it necessary to further explore the role of TUBB in cancer (Alhammad, 2022; Shao et al., 2022; Zhu et al., 2024). To further verify the potential druggability of *TUBB* in DLBLC, we used the small-cell inhibitor targeting TUBB (ABT-751) in *ex vivo* experiments. The results showed that treatment with 1 μM ABT-751 led to a time-dependent inhibition on the expression of β-Tubulin in U2932 cells and SU-DHL-6 cells (Figure 6A), and also promoted the expression of cleaved caspase-3 and significantly increased the apoptotic fraction in both two DLBCL cell lines (Figure 6B), suggesting the anti-DLBCL potential of targeting TUBB.

Subsequently, we performed bulk RNA sequencing on two cell lines treated with either DMSO (solvent) or ABT-751 (1 μM) for 24 h, with five samples per group. The results of the principal component analysis (PCA) revealed that the gene expression profiles of both cell lines underwent obvious alterations following ABT-751 treatment, and these changes exhibited high intra-group consistency (Figure 6C). Further differential expression analysis identified differentially expressed genes after ABT-751 treatment, with a notable downregulation of TUBB gene expression in both cell lines (Figure 6D). Pathway enrichment analysis based on Gene Ontology (GO) terms indicated that these differentially expressed genes were predominantly associated with mitosis-related pathways, such as microtubule formation and chromosome segregation, consistent with the function of TUBB (Figure 6E). Additionally, using the gene set variation analysis (GSVA), we calculated enrichment scores for microtubule-related pathways and found that ABT-751 treatment reduced these scores in both cell lines (Figure 6F). Moreover, consistent with the trends observed in the Annexin V apoptosis detection, apoptosis scores calculated using the PROGENy also showed a higher trend in the ABT-751 group, although this increase was not statistically significant in the SU-DHL-6 cell line (Figure 6G).

## Discussion

In recent years, with the advances in molecular biology and genetics, multiple potential therapeutics have been identified for DLBCL, such as targeting B-cell receptor (BCR) signaling pathways and epigenetic modifications that play key roles in the pathogenesis of DLBCL (Al Hadidi et al., 2024; Lowni et al., 2024; Zhao et al., 2023; Zou et al., 2024; Bakhtiyaridovvombaygi et al., 2024). The development of small molecule inhibitors, monoclonal antibodies, cell therapy products, etc., offers new therapeutic possibilities for patients (Yang and Wang, 2023; Kupcova et al., 2024; Yin et al., 2021; Yang et al., 2023). Moreover, with the development of personalized medicine, targeted treatment strategies based on specific genetic backgrounds of patients, such as treatments targeting specific mutations or aberrantly expressed proteins, are being developed and tested (Yang et al., 2024; Gong et al., 2024). These emerging treatment strategies aim to enhance the efficacy of DLBCL treatments, reduce relapses and improve patient survival rates. Therefore, in-depth research on DLBCL and the development of new therapeutic targets are crucial for enhancing treatment outcomes and overcoming relapses.

In the present study, we investigated the novel potential drug targets from the perspective of plasma proteomes connected with large-scale GWAS data. Via both training and validation, a total of 16 genes, 2 cis-proteins and three trans-proteins were identified to be target candidates associated with the risk of DLBCL. The subsequent colocalization analysis revealed that six genes and two proteins shared the causal genetic variants with DLBCL. Meanwhile, we also found that most of these candidate targets had significant risk connections with DLBCL’s risk factors or associated diseases. Moreover, for the candidate genes or proteins identified above, we performed cell type-specific expression analyses using single-cell sequencing data from DLBCL tumors. These analyses suggested that *TUBB* was highly expressed in malignant B cells, with expression levels increasing as the malignancy worsens. The small-molecule drug targeting TUBB also further confirmed its anti-DLBCL ability, suggesting the druggable potentials.

The MR analysis based on large-scale GWAS data has greatly facilitated the discovery of disease causality and drug targets. Previous studies have identified and validated the effectiveness of this method in drug target discovery across various diseases (Chong et al., 2019; Rasooly et al., 2023; Sun et al., 2024; Yarmolinsky et al., 2022). For instance, Xu et al. applied MR to link plasma metabolites and immune traits with DLBCL risk (Xu et al., 2025), while Pan et al. performed proteome-wide MR across hematologic malignancies, identifying established targets like BCL2 and TNFRSF14—the latter also emerging as a candidate in our eQTL analysis, providing convergent genetic support (Pan et al., 2025). Our study aligns with these efforts in leveraging genetic instruments for causal inference; however, it distinguishes itself by integrating both cis- and trans-regulated plasma proteins alongside eQTL-derived gene targets within a unified discovery framework. This approach enabled the identification of a distinct candidate panel including TUBB, APOM, MICB, and IL17F. Furthermore, whereas prior work by Guo et al. emphasized molecular docking for target prioritization (Guo et al., 2025), our experimental validation functionally demonstrates anti-DLBCL efficacy using a specific TUBB inhibitor (ABT-751), corroborated by transcriptomic evidence of downstream pathway perturbation. In summary, our findings both complement and extend existing multi-omics MR studies by offering a distinct, protein-centric perspective that directly links genetic discovery to pharmacological validation in DLBCL. Moreover, further integration of multi-omics, such as plasma proteomics and transcriptomics, has further promoted the development of therapeutic targets with the application of MR. However, due to the reporting lag in large-scale GWAS cohort studies, research using these methods to explore novel drug targets in hematologic malignancies is very scarce. Therefore, for the first time, we utilize two large DLBCL GWAS cohorts to explore drug targets based on plasma proteomes and gene expressions, probably promoting the discovery of new therapeutics for DLBCL.

Although multiple potential therapeutic targets associated with DLBCL risk have been identified, the localization and relationships of these target genes within DLBCL remain unclear. However, pathway enrichment and protein-protein interaction analyses targeting these genes did not reveal any connections between them. Consequently, we further identified cell type-specific expressions in DLBCL patients through single-cell transcriptome data (Sun et al., 2023; Hamel et al., 2024; Hao et al., 2024), finding that TUBB is highly expressed in malignant B cells and positively correlates with their degree of tumor malignancy. As no previous studies have reported the relationship between TUBB and DLBCL, we then used TUBB inhibitors (ABT-751) to validate the anti-DLBCL efficacy of targeting TUBB, further suggesting the potential application of these targets in the future.

The greatest strength of this study is that it is the first to search for new DLBCL targets from the perspective of plasma molecular multi-omics. The study design was characterized by broad data sources, large sample sizes and high coverage of proteomics and transcriptomics. Methodologically, we used independent analyses of training and validation sets, and subsequently verified the reliability of the results through multi-dimensional and multiple methods, providing new insights for the development of new drugs for DLBCL. However, several limitations of this study still need to be considered. Firstly, although multiple potential drug targets for DLBCL were identified, only the efficacy of TUBB was further validated at the single-cell level, *in vitro* experiments, and *in vitro* transcriptomics because of the scope and resource constraints of a single study. Further in-depth exploration of these target genes in DLBCL is needed in the future. Secondly, the GWAS cohorts used in this study were limited to European populations, and whether the discovery of these target genes is applicable to other ethnicities still needs further confirmation. Thirdly, this study is subject to potential biases inherent in public datasets, including unmeasured confounding and selection bias, which may affect the generalizability of the findings. Additionally, Mendelian randomization relies on core assumptions that cannot be fully tested with the available summary-level data. Finally, although we validated TUBB using *in vitro* experiments, functional verification for the remaining candidate targets is still limited and requires further experimental investigation.

In summary, we identified a series of novel proteins or genes associated with DLBCL risk that can potentially be targeted, providing new insights into the development of DLBCL treatment from the perspective of plasma molecular multi-omics. Further preclinical and clinical researches are required to confirm the potential drug-targeting properties and mechanisms of these candidates.

## Data Availability

The datasets presented in this study can be found in online repositories. The names of the repository/repositories and accession number(s) can be found in the article/Supplementary Material.

## Supplementary material

SUPPLEMENTARY FIGURE 1 Pathway enrichment analysis of genes identified by Mendelian Randomization analyses.

SUPPLEMENTARY FIGURE 2 Protein-protein interaction network of genes identified by Mendelian Randomization analyses.

SUPPLEMENTARY FIGURE 3 Violin plots showing the normalized expression levels of representative marker genes for different cell types, with 25th, 50th and 75th percentiles marked.
