# Supplementary figures and images for: Unraveling novel therapeutic targets for diffuse large B-cell lymphoma: a plasma multi-omics study
Source: Front Pharmacol. 2026 Apr 30;17:1833739. doi: 10.3389/fphar.2026.1833739 (PMC13171477; doi:10.3389/fphar.2026.1833739)

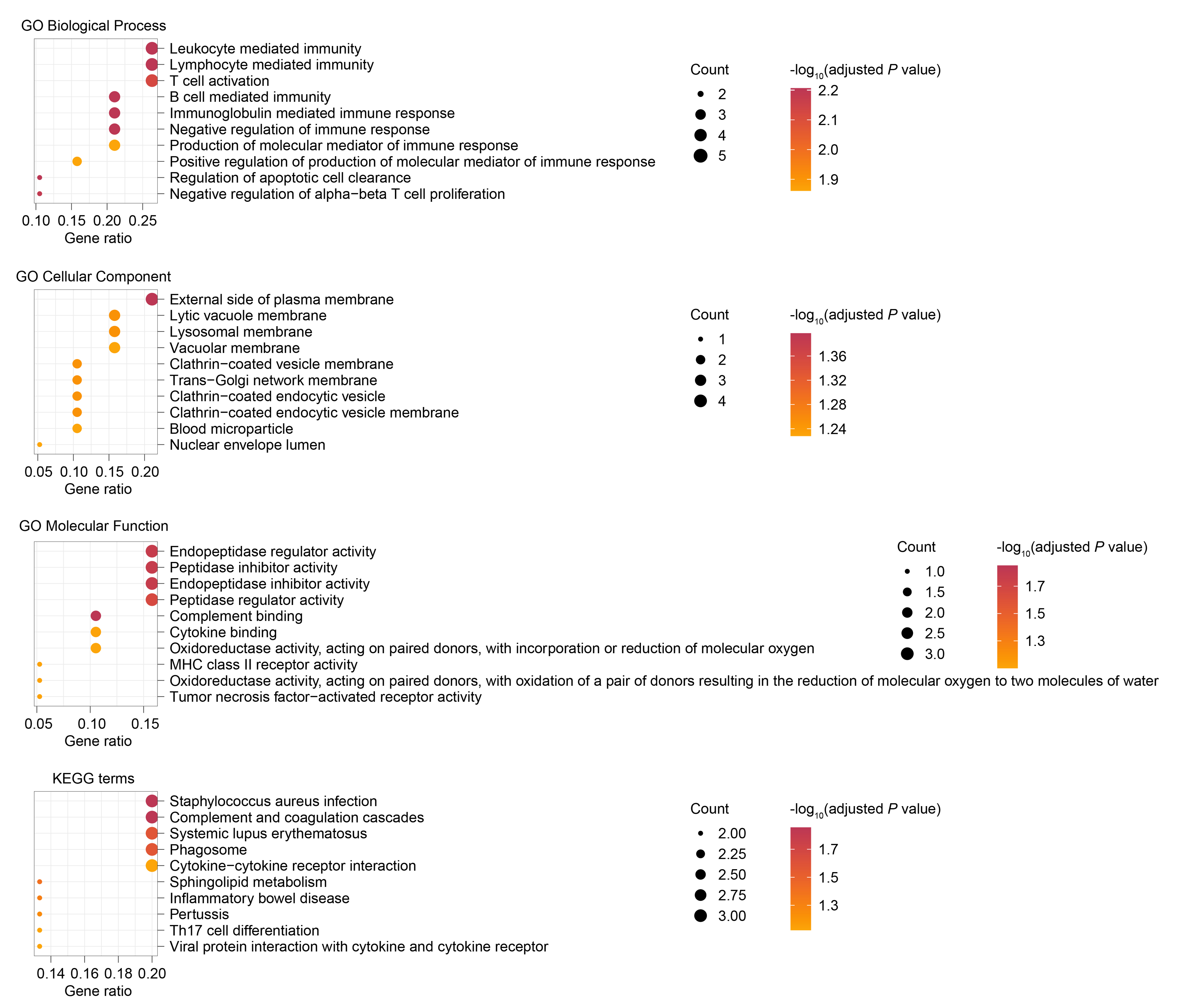

Supplement: Supplementary file 1 [file DataSheet1.zip › Supplemental Figures and Tables/Supplemental Figure/Supplemental Figure 1.jpg]

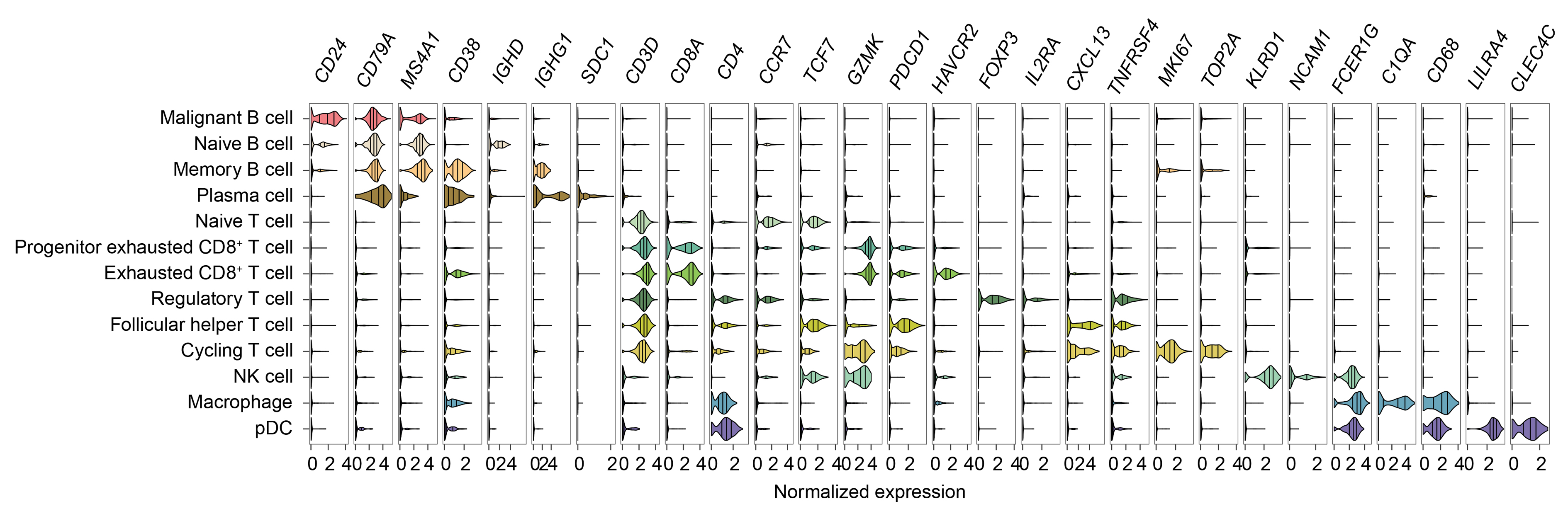

Supplement: Supplementary file 1 [file DataSheet1.zip › Supplemental Figures and Tables/Supplemental Figure/Supplemental Figure 3.jpg]

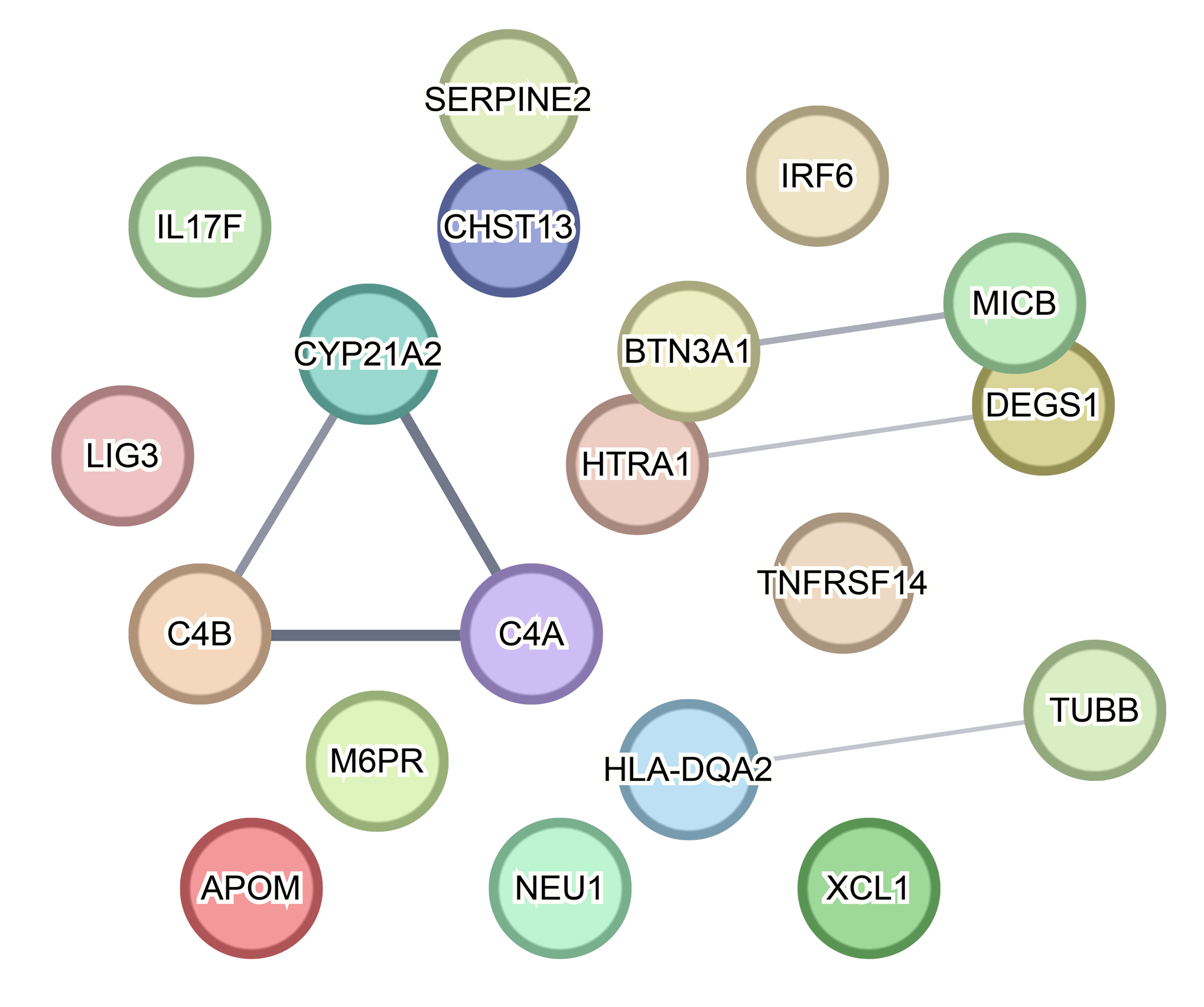

Supplement: Supplementary file 1 [file DataSheet1.zip › Supplemental Figures and Tables/Supplemental Figure/Supplemental Figure 2.jpg]
